# Vertical Distribution and Migration Patterns of *Nautilus pompilius*

**DOI:** 10.1371/journal.pone.0016311

**Published:** 2011-02-22

**Authors:** Andrew J. Dunstan, Peter D. Ward, N. Justin Marshall

**Affiliations:** 1 School of Biomedical Science, University of Queensland, Brisbane, Queensland, Australia; 2 Department of Biology, University of Washington, Seattle, Washington, United States of America; 3 Queensland Brain Institute, University of Queensland, Brisbane, Queensland, Australia; University of Aberdeen, United Kingdom

## Abstract

Vertical depth migrations into shallower waters at night by the chambered cephalopod *Nautilus* were first hypothesized early in the early 20^th^ Century. Subsequent studies have supported the hypothesis that *Nautilus* spend daytime hours at depth and only ascend to around 200 m at night. Here we challenge this idea of a universal *Nautilus* behavior. Ultrasonic telemetry techniques were employed to track eleven specimens of *Nautilus pompilius* for variable times ranging from one to 78 days at Osprey Reef, Coral Sea, Australia. To supplement these observations, six remotely operated vehicle (ROV) dives were conducted at the same location to provide 29 hours of observations from 100 to 800 meter depths which sighted an additional 48 individuals, including five juveniles, all deeper than 489 m. The resulting data suggest virtually continuous, nightly movement between depths of 130 to 700 m, with daytime behavior split between either virtual stasis in the relatively shallow 160–225 m depths or active foraging in depths between 489 to 700 m. The findings also extend the known habitable depth range of *Nautilus* to 700 m, demonstrate juvenile distribution within the same habitat as adults and document daytime feeding behavior. These data support a hypothesis that, contrary to previously observed diurnal patterns of shallower at night than day, more complex vertical movement patterns may exist in at least this, and perhaps all other *Nautilus* populations. These are most likely dictated by optimal feeding substrate, avoidance of daytime visual predators, requirements for resting periods at 200 m to regain neutral buoyancy, upper temperature limits of around 25°C and implosion depths of 800 m. The slope, terrain and biological community of the various geographically separated *Nautilus* populations may provide different permutations and combinations of the above factors resulting in preferred vertical movement strategies most suited for each population.

## Introduction

Knowledge of the diurnal vertical movements may help to further understand feeding, predator avoidance, energy budget and buoyancy equilibration strategies employed by *Nautilus*. The management of *Nautilus* fisheries also requires information on movement patterns to determine fishing effort in relation to population range and distribution.

The diurnal vertical movements of *Nautilus* have been observed in three different populations/localities; Palau [1], [2], Manus Island - Papua New Guinea [3] and near Port Moresby – PNG [4]. The observed behaviour of shallower water (100–150 m) nocturnal depths and deeper (250–300 m) night-time depths was not inconsistent, and vertical movements are currently considered to be from deep daytime resting locations to the relative shallows of 200 m for night feeding activity. It is understandable to think that *Nautilus* may migrate to the daytime sunlit areas of 200 m or shallower to feed under the cover of darkness and return to the dark depths during the day to avoid visual predators, as was the general conclusion from these studies.

Buoyancy equilibration has also been thought to be a factor in vertical movement of *Nautilus*. Previous work by Ward [5] demonstrates that at depths greater than 250 m the osmotic pressure gradient between the siphuncle and shell chambers causes an influx of liquid to the chambers, resulting in negative buoyancy. Only at depths of less than 250 m is it possible for *Nautilus* to remove this liquid and return to a neutrally buoyant state. Chamber flooding at depth would necessitate any deep forays to be followed by a resting and re-equilibration period around 200 m. The data from the Palau study support this argument with consistent relative stasis periods around 240 m. *Nautilus* are also thought to rest by attaching to the substrate [6] and it has been speculated that they could rest in cool deep waters for months following feeding success to conserve energy [4].

The influence of habitat, substrate and biota on vertical movement of *Nautilus* has not been investigated. Previous studies have identified these features of *Nautilus* study sites in Fiji [7], Palau [2], [8], [9] and the Philippines [6], [10] and these factors are investigated in this paper.

The results of this study provide the first indication that more than one pattern of stereotypic diurnal behaviour is present in the genus. The influence of a range of factors may determine specific patterns of vertical movement undertaken by *Nautilus* from different geographically isolated populations.

## Results

### Ultrasonic tracking

Pre-tagging range testing of ultrasonic tags and tracking equipment showed:

VR100 unit detected transmitter signal to 535 m (SD = 25.5 m, n = 5).
VR2 unit detected transmitter signal to 550 m (SD = 32.5 m, n = 5).

**Figure 1:**
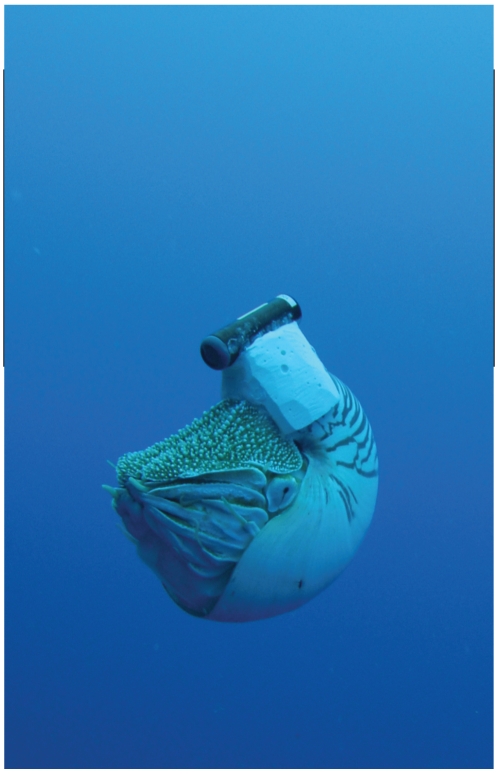
*Nautilus pompilius* with ultrasonic transmitter. *Nautilus pompilius* released at Osprey Reef after tagging with Vemco V16TP transmitter and counter-buoyant epoxy and glass micro-balloon saddle.

**Figure 2:**
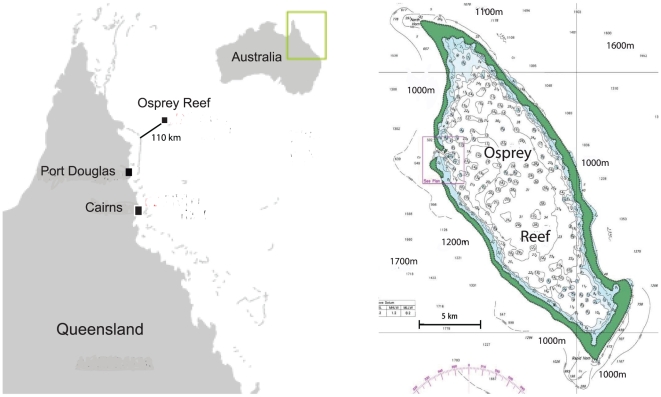
Osprey Reef, Coral Sea, Australia. Location map of the study site, Osprey Reef, showing its position 110 km from the outer edge of the Great Barrier Reef in the Coral Sea. Finer scale bathymetric details highlight the precipitous nature of the reef walls; 1000 m depths within 2 km of the exposed reef, extending to 1700 m depths within 5 km.

Eleven *Nautilus* were tagged with ultrasonic transmitters (Fig. 1) and tracked for up to 78 days (Table S1. Supporting information) at Osprey Reef, Coral Sea, Australia (Fig. 2).

**Figure 3:**
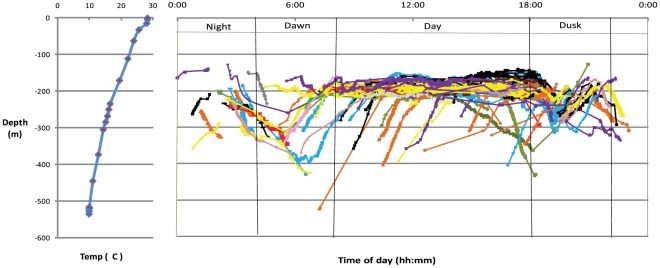
Vertical movements of tagged *Nautilus* with temperature/depth profile. Complete movement data for each individual *Nautilus* (identified in the legend below) are presented in 24 hour periods. Dawn (0400–0800 hrs), Day (0800–1800 hrs), Dusk (1800–2200 hrs) and Night (2200–0400 hrs) periods are labelled. Temperature and depth data are presented from a separate deployment of a Vemco V16TP transmitter not attached to *Nautilus* but recorded during the tracking period. Individual tagged *Nautilus*: #61 (blue diamond); #62 (brown square); #65 (green triangle); #66 (purple cross); #67 (blue asterisk); #68 (orange circle); #69 (purple line); #70 (brown line); #71 (green line); #72 (blue square); #73 (purple diamond).

**Figure 4:**
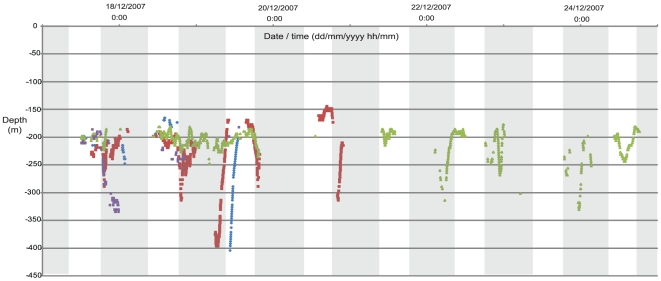
Long term vertical movements of four *Nautilus* individuals. The tracking records of the four *Nautilus* individuals (identified in the legend below) with most continuous monitoring detections are presented. Shaded vertical blocks denote daytime periods (0800–1759 hrs). Individual tagged *Nautilus*: #62 (blue diamond); #65 (brown square); #69 (green triangle); #70 (purple cross).

The resulting data provide the longest and highest resolution (observations/time) record to date of vertical movements of any study of *Nautilus*. Individual tagged *Nautilus* moved large horizontal distances of up to 6 km per day in both directions along the reef face which is consistent with previous observations [11]. Tagged individuals were tracked using four underwater receiver listening stations (VR2 receivers) within a 6 km span of this overall perimeter and four real time tracking sessions (VR100 receiver) of 1–3 days each. The resulting records provide detections of eleven tagged *Nautilus to* 523 m depth throughout all periods of the day and show a consistent pattern of diurnal vertical movement (Fig. 3 & 4).

A consistent trend of daytime (0800–1800 hrs) occurrence at depths of around 200 m was observed.

Dusk (1800–2200 hrs) showed a repeated pattern of descent at around 1800 hrs from daytime depths (DD) of approximately 200 m down to 250–300 m followed by ascent back to DD over a period of two hours before a variety of individual night movements were recorded.

During night periods (2200–0400 hrs) depths ranged between 130–350 m

Dawn (0400–0800 hrs) was the period of deepest movement by *Nautilus*. While not all individuals on each day migrated to >350 m; dawn was the dominant time when depths >350 m were recorded. Excursions deeper than 350 m resulted in a later time of return to the 200 m daytime zone for animals #66, 69, 70 and 71 (Fig. 3).

The patterns of behaviour described above were observed during 82.5% of the dates where movement records of greater than 2 hours duration were obtained. Each individual exhibited high percentage adherence to this pattern with the exception of #61 (Table 1). Aberrations to this behaviour are described below.

**Table 1:** Summary of individual nautilus behaviour over observation period.

ID	# dates significant observations	# dates ‘normal’ behaviour	% ‘normal’ behaviour	# dates ‘unusual’ behaviour
#61	5	2	40	3
#62	16	12	75	4
#65	11	9	82	2
#66	8	7	88	1
#67	5	5	100	0
#68	2	2	100	0
#69	13	11	85	2
#70	15	13	87	2
#71	3	3	100	0
#72	1	1	100	0
#73	1	1	100	0
**Total**	**80**	**66**	**82.5**	**14**

A summary of vertical movement behaviour compared with the behaviour hypothesis presented in this paper (‘normal’ behaviour).

Individual *Nautilus* showed a variety of different behaviours within the overall diurnal pattern observed.

### Deep to shallow ascents during daytime

There were eight instances (#62, 65, 69 and 70) recorded of early afternoon depths of 268–405 m with ascents back to daytime depths of approximately 200 m. These were outside the ‘normal’ pattern seen of very deep dawn dives resulting in a late morning return to 200 m depths (Fig. 4).

### 200 m to shallow ascents during daytime

Only one occurrence of shallow ascents during daytime was recorded; #66 moving from 206 m to 149 m and then back to 219 m.

### Early to mid afternoon descents

#61 showed three instances and #62 one instance of early to mid afternoon descents from daytime depths to 252 m, 433 m, 363 m and 289 m respectively.

### Stationary at 200 m outside daytime periods

Only one occurrence of relative stasis outside daytime periods was observed with #65 from 1958 hrs to 2330 hrs within a depth range of 194 m to 215 m.

### Stationary at depth

The only example of any relatively stationary periods at depth was for #65 in the dawn period of 0552 hrs to 0712 hrs at a depth rage of 371 m to 396 m.

**Figure 5:**
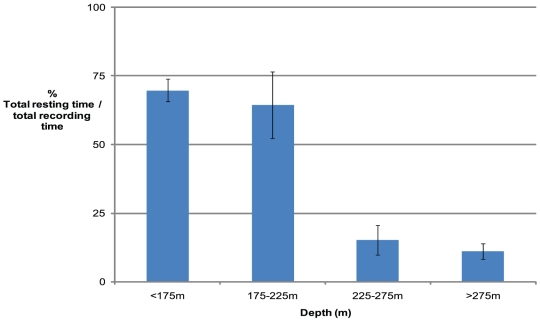
Percentage resting time at depth. Resting periods were compared with total recording time at various depth ranges. Values are total pooled tracking records (error bars are 1 standard error of the mean) from 11 individuals. *Nautilus* movement detections of greater than 5 m were taken as relevant due to depth data errors including minimum tolerance of 4.1 m from both VR100 and VR2W receivers and influence from tidal change and surface movements of the tracking vessel due to wave action. Periods of rest were identified as any time greater than 15 minutes with less than 5 m movement and with detections recorded within at least 15 minutes of each other.

The patterns of resting behaviour of tracked *Nautilus* at various depth regimes within their overall habitat range (Fig. 5) reaffirm the 24 hour trends (Fig. 3) for the entire tracking and detection period of the eleven tagged *Nautilus*.

**Figure 6:**
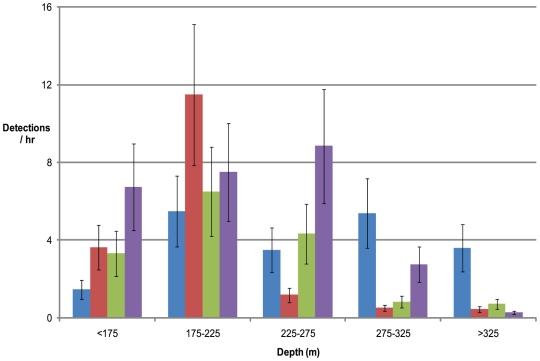
Twenty four hour nautilus depth presence. Data from all animals was pooled to provide an indication of the presence at various depths during dawn, day, dusk and night periods (identified in legend below) from all recordings received. This is presented as number of detections at specific depth ranges per hour of recorded *Nautilus* presence. Values are total detections (error bars are 1 standard error of the mean) from 11 individuals. Time periods: 0400–0759 hrs (blue square); 0800–1759 hrs (brown square); 1800–2159 hrs (green square); 2200–0359 hrs (purple square).

At less than 225 m resting time was 67.1% of total recording time while at >225 m the resting times were minimal (13.1%) and equated with continual movement within these regions consistent with searching for prey (Fig. 6). The Osprey Reef *Nautiluses* are within this <225 m zone most frequently during daytime periods and at depths >225 m during night and dawn times (Fig. 6). Analysis of individual data for the five most recorded *Nautiluses* (#62, 65, 66, 69 and 70) showed exactly the same trend as in the pooled data for percentage resting times. Maximum rate of ascent and descent were calculated for each tagged *Nautilus*. These agree closely with past published data to show maximum *Nautilus* descent and ascent rates to be equivalent at 3.0 m min^−1^ and the average over all individuals to be 2.1 and 2.3 m min^−1^ respectively.

The only periods of relative stasis occurred in depths <235 m. Thirty-six periods of >3 hrs with <25 m vertical movement were recorded in total for all individuals. All of these periods occurred only during daytime (0800–1800 hrs).

All records deeper than 235 m are in ascent or descent mode and the deepest depth is a point of turnaround to immediate ascent. The only example of any relatively stationary periods at depth was for #65 in the dawn period of 0552 hrs to 0712 hrs at a depth rage of 371 m to 396 m.

**Figure 7:**
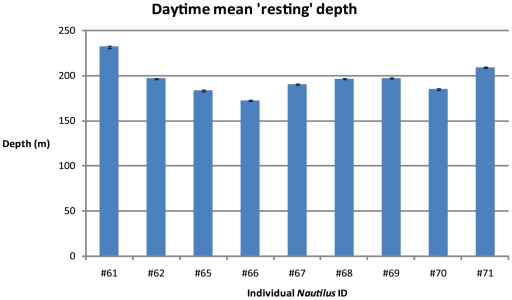
Mean daylight depths of individual nautilus (0800–1800 hrs). Data show mean depths of all individuals for recorded stationary periods of >15 min with less than 5 m vertical movement (error bars are 1 standard error of the mean).

The relatively stationary daytime behavior of all *Nautilus* individuals was similar with a mean depth of 195.8 m±5.7 m. (Fig. 7).

Trapping events showed no captures during daylight hours (n = 10) while night trapping resulted in consistent captures (n = 278±0.35; N = 1559; mean = 5.4 trap^−1^).

### Remotely operated vehicle (ROV) observations

**Figure 8:**
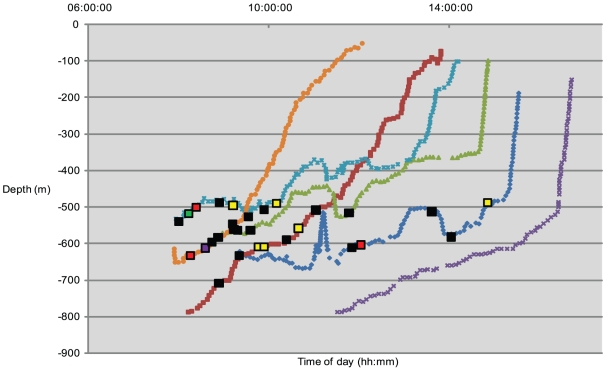
ROV dive profiles and *Nautilus* sightings. The daytime dive profiles for six dives at 4 different sites demonstrate a range of timing for dives. Dive locations are indicated by dark blue diamonds (Coral Castles 1), green triangles (Coral Castles 2), dark brown squares (North Horn), purple crosses (Pavona 1), light blue stars (Pavona 2) and light brown circles (False Entrance). *Nautilus* sightings are identified by large squares of different colors; black (one animal), yellow (two), red (three), green (four) and purple (five). The majority (65%) of sightings were of single individuals. Only one dive, Pavona 1, failed to produce any *Nautilus* sightings.

ROV observations during six dives at four sites on Osprey Reef [12] provided a total of 29.05 hours of video footage and still images from 100 m to 800 m depth during daytime only. Sightings of *Nautilus* were only recorded deeper than 489 m with most between 489–650 m (Fig. 8). All *Nautiluses* were actively moving in foraging mode and many demonstrated some attraction to the ROV. Of the 48 individuals recorded five were juveniles, sighted between 490 m and 608.1 m (Table 2).

**Table 2:** ROV Nautilus sightings at Osprey Reef.

Depth (m)	0–300	300–400	400–500	500–600	600–700	700+	Total
# *Nautilus*	0	0	7	23	17	1	48
# juveniles	0	0	1	3	1	0	5
Search time (hrs)	4.25	3.83	7.92	7.4	4.57	1.08	29.05

ROV dives between 0800–1600 hrs at four sites [12] for a total of 29.05 hours recorded a total of 48 individual *Nautiluses* including five juveniles. All sightings were deeper than 489 m.

**Figure 9:**
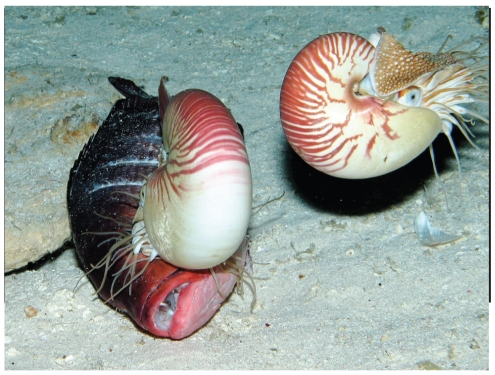
Deepest record of *Nautilus* – 703 meters. *Nautilus pompilius* recorded feeding during daytime at 703 m on red bass bait deposited by an ROV on the sea floor.

At Bougainville Reef a freshly killed red bass (*Lutjanus bohar*) was taken by the ROV down to a depth of 700.5 m and laid out on the sea floor. Four minutes before the bait was laid out a single *Nautilus* approached the ROV. Six minutes after bait positioning two *Nautiluses* came into view in foraging mode. These *Nautiluses* investigated the bait closely and commenced feeding on the fish after a further five minutes (Fig. 9). At post-bait positioning times of 35, 45 and 56 minutes further individual *Nautiluses* arrived.

## Discussion

### Osprey Reef *Nautilus* movements

Wide ranging nightly movements allow *Nautilus* to forage within a depth regime restricted at its shallowest by temperature and at its deepest (800 m) by chamber filling and shell implosion [13], [14]. Observational reports in cooler habitats (New Caledonia and Loyalty Islands) record *Nautilus* as shallow as 5 m during the cooler months of the year while in more equatorial regions the minimum capture depth is around 100 m, consistent with the Osprey Reef data. The limiting maximum temperature for *Nautilus* feeding of around 25°C [3] equates to a depth of 100 m (Fig. 3) which is close to the shallowest of depths recorded during night forays (111.5 m).

The data indicate a strong pattern of vertical movement undertaken by Osprey Reef *Nautilus pompilius*. Daytime is a period of either relative stasis around 200 m depths or deep foraging beneath 489 m, while night-time is an active period of continual movement within the full depth range between 100 to over 700 m. Individuals which remain around 200 m during daytime then undertake a ‘dip’ to 250–300 m at dusk and return to 200 m over a period of approximately two hours before night movement between depths of 110–350 m occurs. Dawn is a time of the deepest records followed by a return to 200 m resting depths or continued feeding at depths >489 m during the day.

Aberrations to this pattern do occur but are infrequent. Daytime descents were seen on four occasions and deep to shallow ascents recorded on eight occasions. Strict adherence to the described pattern may be over-ridden by factors such as hunger which make a feeding foray worth the risk of daytime descent, or buoyancy issues at depth which make ascent essential. Range testing and a maximum recorded tagged *Nautilus* depth of 523 m showed that tagged individuals would be out of range at deeper than around 500 m. The loss of some animals during tracking around dawn could be explained by their movement to greater depths for daytime feeding.

### Comparison with *Nautilus* movements reported from Palau and PNG

**Figure 10:**
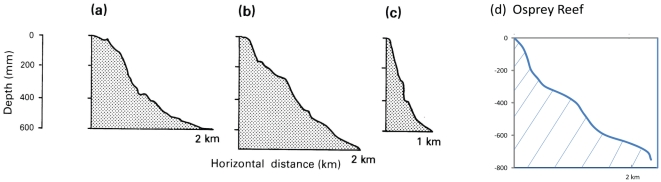
Depth profiles of *Nautilus* habitats. Depth profiles of known *Nautilus* habitats as sourced from Ward [3] and a profile of Osprey Reef at the Coral Castles site. These show very similar step drop-offs on the fore-reef slope to Fiji, Philippines and Palau.

The movements of the sole individual within a 75–250 m zone in O'Dor's PNG study [4] correspond to the findings of this Osprey Reef study. The more detailed studies of Ward and Carlson in Palau [1], [2] are quite different and demonstrate deep water (300–450 m) daytime presence following wide ranging and shallower night activity. The habitat observed here is bathymetrically similar to the three previously studied localities in Palau and PNG and also *Nautilus* habitats described from Fiji and the Philippines (Fig. 10). Diurnal vertical movement differences between this study and those obtained in Palau may be related factors other than simply bathymetry.

### Factors influencing vertical movement patterns – predation, habitat and buoyancy regulation

A combination of factors may determine the vertical movement patterns of *Nautilus* in different geographically isolated populations. Silty and muddy habitat types for optimal feeding may be a key to where *Nautilus* would preferentially spend their time. If the optimal feeding habitat is below 250 m then chamber flooding will create buoyancy regulation tradeoffs; between time availability at depth before a critical level of negative buoyancy is reached and time spent around 200 m for buoyancy re-equilibration. Spending time at 200 m for buoyancy re-equilibration may be impacted by the presence of visual predators, the size of *Nautilus* and available cryptic habitat. In open country with limited hiding options, time spent at 200 m may only be possible during night hours. In the rocky reef habitat of Osprey Reef at 200 m depths and with a relatively small maximum shell size daytime crypsis may be ideal. Whether *Nautilus* will feed throughout the entire day is also crucial to their vertical movement patterns. Previous trapping and aquarium observation reports would suggest that *Nautilus* only feed at night [4], [15] however the data from this study's ROV observations and those of Saunders' Palau photo-sequence study [9] refute this suggestion and provide solid evidence for daytime feeding. From previous population estimation studies of Osprey Reef *Nautilus* [12], around 32% of the population are actively foraging during daytime at depths >489 m [12].

The relative small size of Osprey Reef *Nautilus* (132 mm diameter [16]), compared to the Palau individuals (202 mm diameter [17]) may provide an advantage in daytime crypsis and predator avoidance. The habitat in Palau at 200 m may not provide the necessary hiding places for the larger animals during daylight and it may be a better survival option to spend daylight hours in depths >300 m with lower light levels. This would necessitate spending considerable time during night hours in the shallower 200 m zone to re-establish neutral buoyancy after influx of liquid to chambers during deep daytime periods.

Food availability may also be a major factor in depth movements, where silty or muddy substrate below a reef drop-off provides an optimal feeding zone. These habitat types recorded maximum catch rates at all *Nautilus* locations sampled in the available literature. From ROV observations at Osprey Reef the 100–250 m zone is a steep, rocky reef wall recording low catch rates which becomes a more gradually sloping silty substrate from 300 to 450 meters producing higher catch returns [16]. The Palau photo-sequence images show a similar silty substrate is present in the 150–300 m zone where the greatest number of *Nautilus* were attracted to camera traps while below 350 m only occasional sightings were recorded of mostly single animals [9]. In trapping studies in Fiji, sampling below the steep reef wall at 300 m on muddy substrate provided the best catch rates, while in New Caledonia optimal catches were at 50–75 m on coral, reef talus or coralline sand substrates very near the reef wall [7]. In the Tanon Straits, Philippines with a shallower sloping muddy-silty bottom from 50–320 there was constant catch rate from 61 to 320 m [6]. This zone of maximum catch rates most likely relates to a preferential exposed feeding area characterized by the potential for scavenging dead organisms descended from the reef walls above or preying on crustaceans or their discarded exoskeletons.

Physiological data from previous work on *Nautilus* buoyancy maintenance [5] supports the diurnal behaviour model seen in Osprey individuals as well as the very different behaviour (but with considerable periods around 200 m at night) in the Palau and Manus Island studies. At depths greater than 250 m the osmotic pressure gradient between the siphuncle and shell chambers causes an influx of liquid to the chambers, resulting in negative buoyancy. Only at depths of less than 250 m is it possible for *Nautilus* to remove this liquid and return to a neutrally buoyant state. Consistent observations of *Nautilus* below 450 m during daytime periods may indicate a time period of longer than a single day for chamber filling to reach a critical point which necessitates a return to 200 m for buoyancy re-equilibration. Previous reports [4], [18] speculate that *Nautilus* could rest in cool deep waters for months following feeding success to conserve energy. *Nautilus* are capable of metabolic suppression in anoxia and low temperatures, and can even consume oxygen stored in their shells for buoyancy when necessary [19], but this may only occur in extreme conditions. This data shows no evidence of extended resting periods beyond a single daytime event following night foraging and dispels the theory that *Nautilus* rest by hanging on to coral or within crevices for extended periods. Others have calculated *Nautilus* energy expenditure for vertical movement as equivalent for both ascent and descent [4]; however this was only measured within a 75–250 m depth range. This may be true for neutrally buoyant animals but would not be the case for negatively buoyant *Nautilus* at depths of 350 m plus with an excess of chamber liquid and following a successful feeding foray. Extended deep resting periods would in fact result in increased chamber filling and decreased buoyancy making return to shallower waters a journey of increased energy expenditure through vertically propelled swimming.

It is also important to know whether vertical movement behavior and depth distribution is the same for all individuals within a population. The data from trapping records and telemetry show no difference between males and females or between immature, sub-adult or adult individuals. ROV sighting data provide records of juveniles (200–610 m) within almost the entire range of adult *Nautilus* (100–700 m) of Osprey Reef [12]. This importantly challenges past theories [3] that juveniles occupy a different depth zone or habitat to adults. These observations suggest that habitat partitioning is not occurring and that low numbers of juveniles in capture records, deep camera photo-sequences and in ROV observations may be a correct representation of low juvenile abundance.

### Conclusion

Diurnal vertical migrations of *Nautilus* may well be determined by the individual characteristics of the population and habitat of each regional clade. Small isolated seamounts, large island regions with steep reef drop-offs, extended outer barrier reefs and gently sloping deep channels and straits may provide very different habitat types and influence vertical movement behavior. The factors of preferred feeding habitat, requirement for buoyancy regulation and avoidance of visual predators may combine to create vertical migration patterns best suited to each *Nautilus* population and location.

This movement data will contribute greatly towards the essential population and ecology based knowledge needed by the IUCN to correctly list and preserve *Nautilus*. Fishery exploitation driven by demand from the ornamental shell trade continues to threaten *Nautilus* populations in their limited distribution range and has resulted in major declines in localised populations [6]. Conservation of such a richly iconic group, survivors of six major mass extinction events and almost unchanged through 500 million years, must be a priority.

## Materials and Methods

### Ethics statement

Research was conducted under permit from the Australian Fisheries Management Authority and with ethics approval from the University of Queensland Animal Ethics Committee. ANIMAL ETHICS APPROVAL CERTIFICATE Date: 15-Dec-2008.

Professor Justin Marshall, Biomedical Sciences, Ecology of Nautilus, Approval Duration: 15-Dec-2008 to 15-Dec-2011, AEC Approval Number: SBMS/976/08/ARC LINKAGE.

### Capture of *Nautilus*

The study site, Osprey Reef, is an isolated seamount with an almost vertically walled reef perimeter of approximately 70 km. Eleven *Nautilus pompilius* (10 mature males and 1 immature female) of mean 131.5 mm diameter (SD = 6.9 mm) were trapped from depths of 250–300 m off the edge of Osprey Reef as previously described [20] and tracked for up to 78 days (Table S1).

### Ultrasonic tagging of individuals

Detection ranging checks for ultrasonic tracking equipment were performed by suspending transmitters at 10 m depths at a fixed open water point. Both VR100 with omni-directional hydrophone and VR2 receiver units were then suspended below a vessel which was moved increasing distances from three transmitters and GPS (Garmin GPSMAP 276C ) positions and time recorded relative to the fixed transmitter position. Records were taken at approximately 25 m intervals until detection was no longer possible.

Differential pressure transmitters (Vemco, Nova Scotia, model V16P) were attached with buoyant glass micro-balloon/epoxy saddles, to counteract the in-water weight of the transmitter, and then released back into these same depths. The transmitters signal provided both location and depth data. Tracking of animals was conducted from an inflatable dinghy using a Vemco VR100 receiver with omni-directional hydrophone during four real time periods of from one to three days following release. Detection of tagged individuals was discontinuous as animals moved out of range of VR2W receivers and the VR100 unit was moved between widely ranging individuals. Remote tracking was conducted for the three month life of transmitters using fixed VR2W receivers positioned 20 metres underwater at four sites within 6 km of the release area. Detailed vertical movement data for the tagged *Nautilus* were obtained through real time tracking while more sporadic records were obtained when tagged animals were within approximately 500 m of the underwater receiver stations.

### Analysis of tracking data

Analysis of the data for the first 24 hours of tracking showed individuals to settle into their longer term behaviour patterns within the first twelve hours after release and descent. For this reason the first 12 hours of data after release are not used in the analysis of results. Data from both methods were found to be consistent showing time and depth to correlate precisely and allowing data sets to be combined in all subsequent calculations and results. Detection records for analysis of behaviour were restricted to all detections within 15 minutes of each other and with >two hours of detection periods within a 24 hour period (Table 1). For *Nautilus* depth distribution over 24 hours all detections were used (Fig. 2, 3 & 4). *Nautilus* movement detections of greater than 5 m were taken as relevant due to depth data errors including minimum tolerance of 4.1 m from both VR100 and VR2W receivers and influence from tidal change and surface movements of the tracking vessel due to wave action. Periods of rest were identified as any time greater than 15 minutes with less than 5 m movement and with detections recorded within at least 15 minutes of each other (Fig. 5 & 6). Horizontal positions were gained through GPS readings from the VR100 unit and known positions of VR2W receivers. Temperature and depth data are presented from a separate deployment of Vemco V16TP transmitter not attached to *Nautilus* but recorded during the tracking period.

### Remotely operated vehicle (ROV) observations

A Cherokee ROV capable to 800 m depths was deployed at Osprey Reef for six daytime observations at four different sites for dives of between four to eight hours [12]. Standard mini DV footage and still images using white halogen light sources were taken alongside real-time linked observer data recording. Records of depth and location of the ROV were then matched with image and written records for nautilus presence and maturity.

## Supporting Information

Table S1 **Depth and detection summary for tagged *Nautilus***
(DOCX)
