# Supplementary material for: Vertical Distribution and Migration Patterns of Nautilus pompilius
Source: PLoS One. 2011 Feb 22;6(2):e16311. doi: 10.1371/journal.pone.0016311 (PMC3043052; doi:10.1371/journal.pone.0016311)
Supplement: Table S1 — Depth and detection summary for tagged Nautilus (DOCX) [file pone.0016311.s001.docx]

**Table S1. Depth and detection summary for tagged *Nautilus***

|  | **#61** | **#62** | **#65** | **#66** | **#67** | **#68** | **#69** | **#70** | **#71** | **#72** | **#73** |
| --- | --- | --- | --- | --- | --- | --- | --- | --- | --- | --- | --- |
| **Sex** | M | M | M | M | M | F | M | M | M | M | M |
| **Shell diameter (mm)** | 136 | 141 | 140 | 130 | 125 | 117 | 130 | 135 | 134 | 127 | 131 |
| **Maturity** | M | M | M | M | M | I | M | M | M | M | M |
| **min. depth (m)** | 128.0 | 123.8 | 144.5 | 140.4 | 165.1 | 181.6 | 144.5 | 128.0 | 169.3 | 111.5 | 161.0 |
| **max. depth (m)** | 433.5 | 524.3 | 429.3 | 359.2 | 268.3 | 346.8 | 425.2 | 375.7 | 371.5 | 247.7 | 334.4 |
| **Signal interval (s)** | 30 | 30 | 30 | 30 | 30 | 30 | 30 | 30 | 60 | 60 | 60 |
| **# detections*** | 291 | 1079 | 836 | 714 | 182 | 194 | 1119 | 754 | 206 | 104 | 107 |
| **# dates detected** | 5 | 19 | 16 | 8 | 7 | 2 | 20 | 26 | 4 | 2 | 2 |
| **tracking time** | 16 | 49 | 78 | 9 | 13 | 34 | 41 | 58 | 8 | 2 | 2 |
| **(days)** |  |  |  |  |  |  |  |  |  |  |  |

The detection level, frequency and tracking time for each tagged *Nautilus* as well as its maximum and minimum detected depths are summarised. Maturity was based on the presence of an apertural black line in mature animals (M) or absence in immature specimens (I). Signal interval is the mean time between transmitter signal transmissions. Detections were those recorded by either VR100 or VR2W receivers. Tracking time is from release until last detection.
